# Evaluation of the Biostimulant Activity of Zaxinone Mimics (MiZax) in Crop Plants

**DOI:** 10.3389/fpls.2022.874858

**Published:** 2022-06-16

**Authors:** Jian You Wang, Muhammad Jamil, Md. Golap Hossain, Guan-Ting Erica Chen, Lamis Berqdar, Tsuyoshi Ota, Ikram Blilou, Tadao Asami, Samir Jamil Al-Solimani, Magdi Ali Ahmed Mousa, Salim Al-Babili

**Affiliations:** ^1^The Bio Actives Lab, King Abdullah University of Science and Technology, Thuwal, Saudi Arabia; ^2^Center for Desert Agriculture, King Abdullah University of Science and Technology, Thuwal, Saudi Arabia; ^3^Department of Arid Land Agriculture, Faculty of Meteorology, Environment and Arid Land Agriculture, King Abdulaziz University, Jeddah, Saudi Arabia; ^4^Plant Science Program, Biological and Environmental Science and Engineering Division, King Abdullah University of Science and Technology, Thuwal, Saudi Arabia; ^5^Applied Biological Chemistry, The University of Tokyo, Bunkyo City, Japan; ^6^The Laboratory of Plant Cell and Developmental Biology, King Abdullah University of Science and Technology, Thuwal, Saudi Arabia; ^7^Department of Vegetables, Faculty of Agriculture, Assiut University, Assiut, Egypt

**Keywords:** biostimulant, apocarotenoids, zaxinone, zaxinone mimics (MiZax), green pepper (*Capsicum annuum*), squash (*Cucurbita pepo*), tomato (*Solanum lycopersicum*), date palm (*Phoenix dactylifera*)

## Abstract

Global food security is a critical concern that needs practical solutions to feed the expanding human population. A promising approach is the employment of biostimulants to increase crop production. Biostimulants include compounds that boost plant growth. Recently, mimics of zaxinone (MiZax) were shown to have a promising growth-promoting effect in rice (*Oryza sativa*). In this study, we investigated the effect of MiZax on the growth and yield of three dicot horticultural plants, namely, tomato (*Solanum lycopersicum*), capsicum (*Capsicum annuum*), and squash (*Cucurbita pepo*) in different growth environments, as well as on the growth and development of the monocot date palm (*Phoenix dactylifera*), an important crop in the Middle East. The application of MiZax significantly enhanced plant height, flower, and branch numbers, fruit size, and total fruit yield in independent field trials from 2020 to 2021. Importantly, the amount of applied MiZax was far less than that used with the commercial compound humic acid, a widely used biostimulant in horticulture. Our results indicate that MiZax have significant application potential to improve the performance and productivity of horticultural crops.

## Introduction

Ensuring food security is a global issue challenged by different factors, such as climate change, environmental pollution, and, particularly, the rapid growth of the human population (Shaheen et al., 2017). According to the report of the United Nations Food and Agriculture Organization (FAO), food production must increase to double to feed the ever-increasing human population around the world by 2050 (FAO: The World Needs 70% More Food by 2050).^1^ Inarguably, enhancing the yield of crops is one of the solutions to fulfill the goal. Naturally, the growth of crop plants relies on their ability to get nutrients from the soil, which is inevitably affected by unfavorable growth conditions, such as drought, salinity, or biotic stresses (Parida and Das, 2005; Takahashi et al., 2020; Iqbal et al., 2021). Usually, these conditions negatively affect the plant’s health and development, which could reduce the final yield (Pandey et al., 2017). Moreover, climate changes decrease the green land area for food production, while the increase of freshwater usage of the human population also influences crops irrigation (Zhao et al., 2017; Raza et al., 2019). To minimize these adverse impacts, one of the strategies to help the plants to overcome unfavorable growth problems is the usage of biostimulants, which accelerate not only plants’ life cycle, but also maximize fruit production (Bulgari et al., 2015).

**FIGURE 1:**
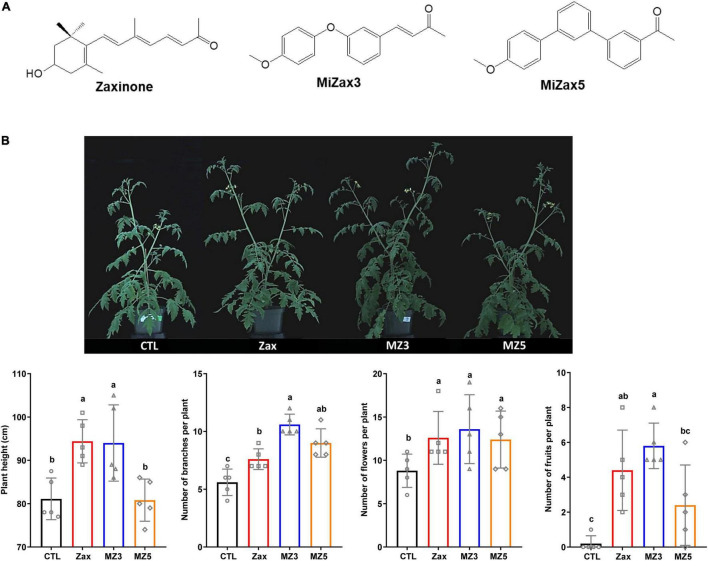
**(A)** Chemical structures of zaxinone, MiZax3, and MiZax5. **(B)** Foliar application of Zax, MZ3, and MZ5 at 5 μM on tomato plants under greenhouse conditions in 2019. Each data point represents one plant (*n* = 5). Data represent mean ± SD. Statistical analysis was performed using one-way ANOVA and the Tukey’s *post hoc* test. Different letters denote significant differences (*p* < 0.05). Symbols (○, CTL; □, Zax; △, MiZax3; and ⋄, MiZax5) used over error bars represent each replicates in the investigated group. CTL, control; Zax, zaxinone; MZ3, MiZax3; MZ5, MiZax5.

Biostimulants are recognized as compounds that enhance plant growth and performance (Du Jardin, 2015; Yakhin et al., 2017), including the specialized metabolites from carotenoid biosynthesis. Carotenoids are isoprenoid pigments that provide precursors for the evolutionary-conserved plant hormones such as abscisic acid (ABA) and strigolactones (SLs) (Al-Babili and Bouwmeester, 2015; Hou et al., 2016; Wang et al., 2021a), as well as apocarotenoid signaling molecules, such as anchorene and zaxinone (Fiorilli et al., 2019; Jia et al., 2019; Wang et al., 2019; Ablazov et al., 2020). Due to the instability of authentic metabolites or restricted natural sources, several ABA and SL analogs have been developed in the past few years and some of them have been tested in the field (Samejima et al., 2016; Screpanti et al., 2016; Jamil et al., 2019, 2020, 2022a,b; Kountche et al., 2019; Vaidya et al., 2019). The further case is zaxinone, a candidate of novel apocarotenoid-derived phytohormones, which is required for normal rice growth and development (Wang et al., 2019). Zaxinone (chemical structures shown in Figure 1A) exerts its function likely through promoting sugar metabolism and regulating the homeostasis of SLs and cytokinins in rice roots (Wang et al., 2019, 2021b). In addition, the application of zaxinone increased wild-type rice (*Oryza sativa*) performance, which indicated its biostimulant activity (Wang et al., 2019). Therefore, we have developed mimics of zaxinone (MiZax), which were supposed to be tested and used as biostimulants. After screening a number of mimics, we identified MiZax3 and MiZax5 (chemical structures shown in Figure 1A) as two compounds exerting similar biological activities of zaxinone with respect to rice grown hydroponically and in the soil systems (Wang et al., 2020). However, the growth-promoting activities of MiZax on the horticultural crops still remain elusive. It is plausible that introducing these biostimulants might bring a positive impact on yield, quality, and overall economic benefit to horticultural crops.

In this report, we tested zaxinone and MiZax on tomato [*Solanum lycopersicum* (*S. lycopersicum*)] plants under greenhouse conditions by foliar application. Moreover, to extend its practical application, we conducted four independent open field studies with three different fruit crops, namely, green pepper [*Capsicum annuum* (*C. annuum*)], squash [*Cucurbita pepo* (*C. pepo*)], and date palm [*Phoenix dactylifera* (*P. dactylifera*)] during the planting year 2020–2021 in the desert climate of Kingdom of Saudi of Arabia. Application of MiZax significantly increased the plant height and branches of date palm, as well as the flower numbers, fruit size, and total fruit yield of green pepper and squash. Notably, we observed these effects with MiZax amounts that are far less than that used for the commercial biostimulant humic acid, a common used growth regulator with positive effects on sustainable agriculture (Canellas et al., 2015 and references therein). Our results indicate that MiZax are potential biostimulants promoting the growth and yield of horticultural plants under different environmental conditions and, hence, they contribute to ensuring global food security.

## Materials and Methods

### Plant Material and Growth Conditions

Tomato seeds (*S. lycopersicum cv*. Moneymaker) were sown in a 24-well plastic tray to raise nursery. 1-week-old uniform seedlings were then transferred to 3 L plastic pots. Each pot was sprayed with biostimulants at 5 μM concentration twice per week. The pots were irrigated with nutrient solution when needed. Data on tomato growth, yield, and yield components were collected.

For raising seedlings, green pepper seeds (*C. annuum* L. var. California Wonder) were sown on 10 October 2019 in Jiffy-7 peat pellets (44 mm) containing a peat-based substrate (peat moss) (manufactured by Norway). Before sowing, seeds were soaked into water overnight and pelleted for 2 h. Pellets were then placed into the trays with seeds into hole and covered very well. After the appearance of first true leaves, plants were watered as required. After 5 weeks, the seedlings were transplanted into the well-prepared plot to initiate the experiment.

Squash (*C. pepo*) seeds of the variety Camila 625 F1 Hybrid (Emerald Seeds Company, United States) were obtained from the local agricultural market at Jeddah, Saudi Arabia.

### Field Trials at the King Abdulaziz University Station

The field experiments were conducted at the Agricultural Research Station, Hada Al-Sham (21°48′3″ N, 39y43′25″ E), King Abdulaziz University (KAU), Jeddah, Saudi Arabia to evaluate the performance of plant biostimulants (zaxinone and MiZax) and humic acid applications on growth and yield of green pepper under freshwater and salty water and squash under freshwater. The soil texture of the experimental site was classified as sandy loam, soil pH 7.8, and EC 1.79 d Sm^–1^. The dominant climate of the area is arid, with high temperatures and long photoperiods during summer season (Mousa and Al-Qurashi, 2018).

Directing seeding was used to plant the squash seeds with the distance between rows of 1 m and 0.5 m between each two adjacent plants in the same row. The squash plants were irrigated with low saline water (3.51 dSm^–1^) until end of growing season. The squash experiment followed a randomized complete block design (RCBD) with 3 replicates. Seedling of the green peppers were planted in plots each of 2.8 m × 1.8 m and the planting distance was 70 cm × 60 cm. The experiment was laid on a split-plot design where three levels of water salinity were assigned to the main plots, while mimics of zaxinone (MiZax3 and MiZax5) and humic acid were randomly distributed over the subplots following RCBD with 3 replicates. The water salinity of underground water was 3.51 d Sm^–1^ and used as control treatment. NaCl was added weekly to the other tanks to raise the water salinity to 8.04 and 11.71 d Sm^–1^ during the season. For watering squash and green peppers, a drip irrigation system was instilled to supply the plants with their water requirements twice per day for 10 min. The plants were weekly fertilized with nitrogen, phosphorus, and potassium (NPK) fertilizer (20:20:20) during vegetative growth and 10:10:40 (N:P:K) at flowering and fruit setting and maturity stages. The biostimulants MiZax3 and MiZax5 at 5 and 10 μM and humic acid at 1 and 1.5 g L^–1^ were foliar applied once per week and for 8 weeks to the plants and after 15 days of transplantation for green peppers and at the second true leaves for squash. Two control treatments received either acetone (as MiZax3 and MiZax5 dissolvent) at 1 ml per L or only water (dissolvent of humic acid). All the recommended agriculture practices for both the crops were applied and kept uniform across the treatments throughout the growing season. The growth and yield parameters of squash, including plant height (cm), numbers of leaves per plant, fresh and dry mass per plant (g), sex ratio (male: female), fruits per plant, and yield per plant (kg), were assessed. For green peppers, we measured plant height (cm) after 20, 40, and 60 days of transplanting, branches, leaves, and flowers per plant at end of season, roots length (cm) and mass (g), plant fresh and dry mass (g), fresh fruits per plant, and yield of fresh fruits per plant (kg). Pepper fruits quality were measured, including weight of single fruit (g), fruit length and diameter (mm), total soluble solid (TSS) (%), vitamin C (mg g^–1^), firmness (N), acidity (EC), and total phenol (mg g^–1^). Soil plant analysis development estimations were taken utilizing SPAD meter (Konica Minolta 502, Tokyo, Japan) from 9:00 to 11:00 a.m. in fully extended third to fifth leaf.

### Field Trial of the King Abdullah University of Science and Technology Experimental Station

A field experiment at the King Abdullah University of Science and Technology (KAUST) (22°18’08.6″N, 39°06’40.0″E) was conducted to evaluate the growth-promoting activity of MZ3 and MZ5 on green pepper (*C. annuum* L. var. California Wonder). After three successive plowing, the field was divided into plots and treatments were allocated by following randomized complete block design (RCBD) with three replications. The plot size was 3 m × 1.2 m and the planting distance was 50 cm × 60 cm. 4-week-old uniform-sized green pepper seedlings were transferred (8 plants per plot) into the field. MiZax3 and MiZax5 were sprayed at 5 and 10 μM concentration with 1 week interval up to 8 weeks. Humic acid was applied at 1 g l^–1^ and untreated plots were included to compare the treatments effect. The plots were irrigated and fertilized when needed and all the other agronomic practices were adopted uniformly by following the standard procedures. Data on plant growth, yield, and yield components were collected.

The field experiment of date palm (*P. dactylifera cv*. ajwa) was conducted at the KAUST study field to investigate the effect of zaxinone and MiZax. Compounds were dissolved in acetone and prepared in a final volume mixed with 0.5% Tween-20. Zaxinone, MiZax3, and MiZax5 were sprayed at 5 μM concentration once per week on the 8-week-old uniform date palm plants. The pots were irrigated with nutrient solution once per week. Data on the shoot growth, branches, and leaves opening were collected.

### Statistical Analysis

Data are represented as mean and their variations as SD. The statistical significance was determined by one-way ANOVA with the Tukey’s multiple comparison test, using a probability level of *p* < 0.05 or the two-tailed Student’s *t*-test to denote significant differences (*p < 0.05, **p < 0.01, ***p < 0.001, ****p < 0.0001). All the statistical elaborations were performed using GraphPad Prism version 8.3.0.

## Results and Discussion

### Zaxinone and Mimics of Zaxinone Enhanced Tomato and Date Palm Plant Growth

Zaxinone and its mimics MiZax3 and MiZax5 showed to promote the growth on the monocot plant rice (Wang et al., 2020). Hence, we hypothesized that these compounds might show the same activity in dicots. To test this hypothesis, we sprayed 5 μM of each of the three stimulants, namely, zaxinone, MiZax3, and MiZax5, to 3-week-old tomato plants for 8 weeks in the greenhouse. Interestingly, we observed striking growth-promoting activities of MiZax3 on plant height, branching, number of flowers, and fruits (Figure 1B). Zaxinone and MiZax5 showed a similar, but less pronounced effects, compared to MiZax3.

**FIGURE 2:**
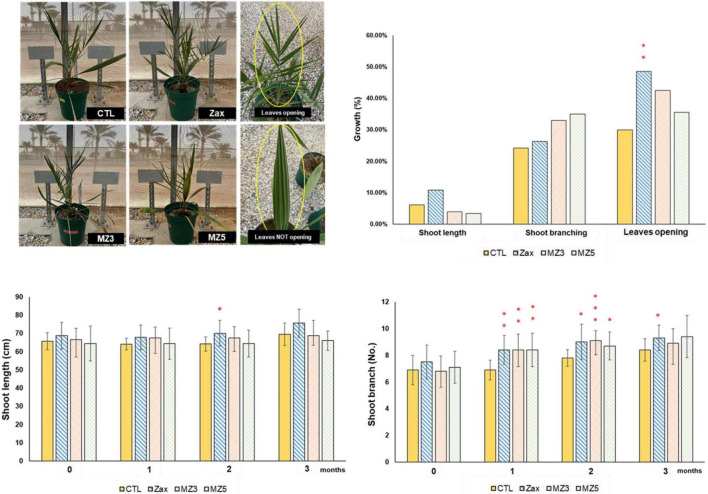
Effect of foliar application of Zax, MZ3, and MZ5 at 5 μM on date palm plants under open field condition, performed in 2021. Data represent mean ± SD. *n* = 10. Statistical analysis was performed using the two-tailed Student’s *t*-test. Asterisks indicate statistically significant differences as compared to CTL (*p < 0.05, **p < 0.01, ***p < 0.001). CTL, control; Zax, zaxinone; MZ3, MiZax3; MZ5, MiZax5.

Next, we applied the three stimulants to plantlets of date palm, an important regional fruit plant for sustaining current desert agriculture in the Middle East (Xiao et al., 2019), to investigate their growth-triggering effect on a monocot tree. Although date palms are slow-growing plants, zaxinone remarkably increased the number of branches, plant height, and leaves opening after 2 months of treatment, while MiZax3 and MiZax5 tended to promote growth (Figure 2). Moreover, these compounds seemed continuously to promote shoot branches at least 1 month after the final application, especially zaxinone led to more leaves opening (Supplementary Figure 1).

These results revealed that the application of MiZax and zaxinone in dicot crop plants and the monocot tree date palm has the same bioactivity as in the cereal plant rice. However, zaxinone was also shown to be a positive regulator on SL and ABA biosynthesis in the dicot plant *Arabidopsis* (Ablazov et al., 2020), which may inhibit root growth and development and indicate that the effect of this compound may differ depending on the plant species. Indeed, both the tomato and date palm contain *ZAXINONE SYNTHASE* (*ZAS*) gene (s) and can form a symbiosis with arbuscular mycorrhizal fungi (AMF). In contrast, *ZAS* is missing in most of the non-mycorrhizal plant, such as *Arabidopsis thaliana* (Wang et al., 2019), which indicates a role of *ZAS* in regulating growth as well as in AM symbiosis. A further point that should be considered is the method of application. Foliar application may lead to different effects, compared with adding the compound to the medium in hydroponic culture as it was done with *Arabidopsis* (Ablazov et al., 2020). Our results also indicate that MiZax3, MiZax5, and zaxinone may differ in particular activities. In summary, MiZax3 and MiZax5 are good candidates for new biostimulants (Yakhin et al., 2017) that can be used to boost tomato and rice plant performance and increase the growth of date palm plantlets under controlled conditions.

### Field Application of Boosted Growth and Yield of Green Pepper Under in Normal Environment and Salty Soil

**FIGURE 3:**
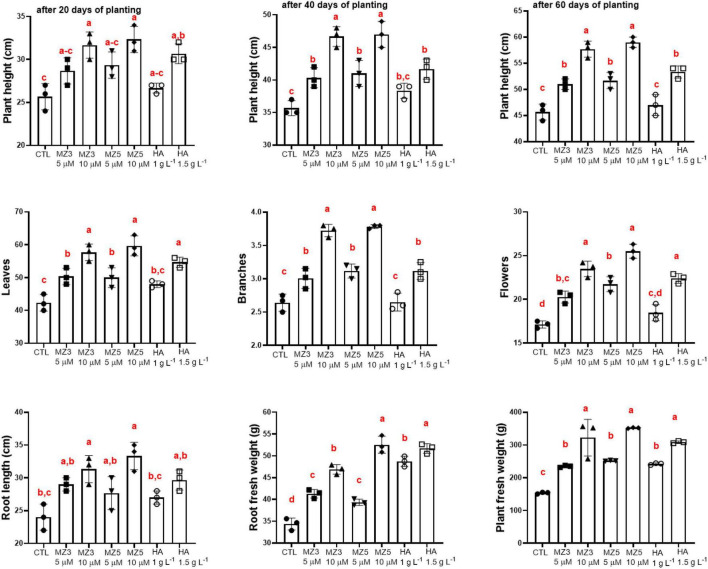
Plant phenotypical evaluation of mimics of zaxinone (MiZax) effect on green pepper from the field of the King Abdulaziz University (KAU), performed in 2020. Each data point represents the average from 15 plants of three plots (*n* = 3). Data represent mean ± *SD*. Statistical analysis was performed using one-way ANOVA and the Tukey’s *post hoc* test. Different letters denote significant differences (*p* < 0.05). Symbols (•, CTL; ■, 5 μM MZ3; ▲, 10 μM MZ3; ▼, 5 μM MZ5; ◆, 10 μM MZ5; ○, 1 g l^–1^ HA; and □, 1.5 g l^–1^ HA) used over error bars represent each replicates in the investigated group. CTL, Control; HA, humic acid; MZ3, MiZax3; MZ5, MiZax5.

To explore the possibility of using MiZax on field application, we tested two concentrations (5 and 10 μM) of MiZax3 and MiZax5 on the valuable crop, green pepper, in comparison to the commercial compound humic acid. We observed that MiZax significantly triggered shoot growth from 20 to 60 days after planting in the Agricultural Research Station of the King Abdulaziz University (KAU) (Figure 3) and clearly boosted the development of flowers, leaves, and branching (Figure 3) in a concentration-dependent manner. Moreover, the root length, root biomass, and plant fresh weight of pepper plants were also increased after MiZax and humic acid application (Figure 3). Surprisingly, MiZax showed, at a concentration of at 5 μM, a comparable effect to 1.5 g l^–1^ of humic acid. Moreover, the performance of MiZax increased, when applied at 10 μM concentration. These positive results were also observed in salty conditions under which MiZax maintained and increased the development of pepper plants (Supplementary Figure 2), indicating that MiZax also increased salt tolerance.

**FIGURE 4:**
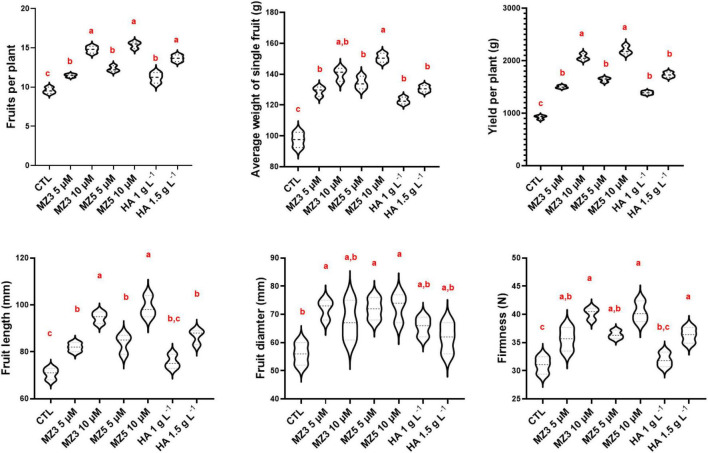
MiZax effect on green pepper fruit production from the field of the KAU, performed in 2020. The data represents as the distribution of 15 plants from three plots (*n* = 3). Data represent mean ± *SD*. Statistical analysis was performed using one-way ANOVA and the Tukey’s *post hoc* test. Different letters denote significant differences (*p* < 0.05). CTL, Control; HA, humic acid; MZ3, MiZax3; MZ5, MiZax5.

**FIGURE 5:**
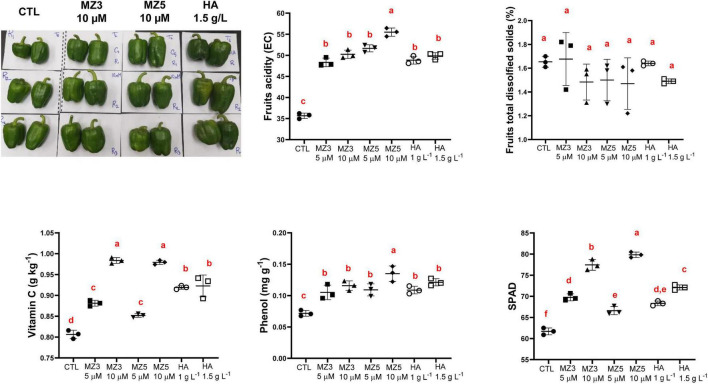
Biochemical analysis of fruits upon MiZax treatment on green pepper from the field of the KAU, performed in 2020. Represented harvested fruits from CTL, 5 μM MZ3, 5 μM MZ5, and 1.5 g l^–1^ HA application were shown here. Each data point represents the average from 15 plants of three plots (*n* = 3). Data represent mean ± *SD*. Statistical analysis was performed using ANOVA and the Tukey’s *post hoc* test. Different letters denote significant differences (*p* < 0.05). Symbols (•, CTL; ■, 5 μM MZ3; ▲, 10 μM MZ3; ▼, 5 μM MZ5; ◆, 10 μM MZ5; ○, 1 g l^–1^ HA; and □, 1.5 g l^–1^ HA) used over error bars represent each replicates in the investigated group. CTL, Control; HA, humic acid; MZ3, MiZax3; MZ5, MiZax5.

Consistent with the observation from plant physiological results about growth and development, the application of MiZax enhanced fruit production and total yield (Figure 4). In addition, the length and width, as well as the firmness of fruits were also enhanced, compared with the control group (Figure 4), even under salty fields, except the diameter under high salt condition (Supplementary Figure 3). To assess the quality of fruits harvested in the KAU field, we measured the content of vitamin C and phenols and quantified the acidity and total dissolved solids. As shown in Figure 5 and Supplementary Figure 4, except for the total dissolved solids, all the biochemical parameters were increased in all the treatments. Although there was no superior difference between MiZax and humic acid, the amount of MiZax used was less than that of humic acid (∼3.5 vs. 1 g l^–1^), which represents a big advantage of MiZax. Moreover, the leaf single-photon avalanche diode (SPAD) values were higher after applying the high concentration of MiZax treatments (Figure 5 and Supplementary Figure 4). This result indicates that MiZax could increase the levels of chlorophyll and might enhance the photosynthetic activities, probably by increasing leaf numbers. A positive effect of zaxinone on photosynthesis in rice was recently reported (Wang et al., 2021b). Moreover, the increased fruit size suggests that MiZax might also enhance source-to-sink allocation, as zaxinone triggers primary metabolism in rice plants (Wang et al., 2021b).

**FIGURE 6:**
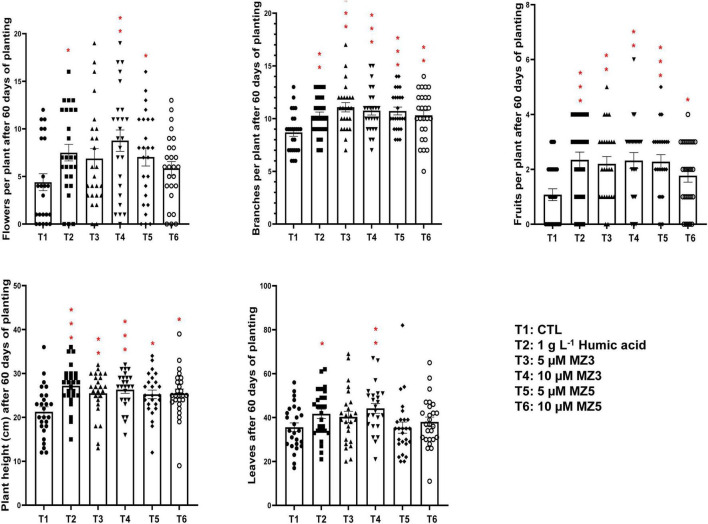
Plant phenotypical evaluation of MiZax treatment on green pepper from the King Abdullah University of Science and Technology (KAUST) field, performed in 2021. Each data point represents one plant (*n* ≥ 25). Data represent mean ± *SD*. Statistical analysis was performed using the two-tailed Student’s *t*-test. Asterisks indicate statistically significant differences as compared with CTL (*p < 0.05, **p < 0.01, ***p < 0.001). Symbols (•, CTL; ■, 1.5 g l^–1^ HA; ▲, 5 μM MZ3; ▼, 10 μM MZ3; ◆, 5 μM MZ5; and ○, 10 μM MZ5) used over error bars represent each replicates in the investigated group. CTL, Control; HA, humic acid; MZ3, MiZax3; MZ5, MiZax5.

**FIGURE 7:**
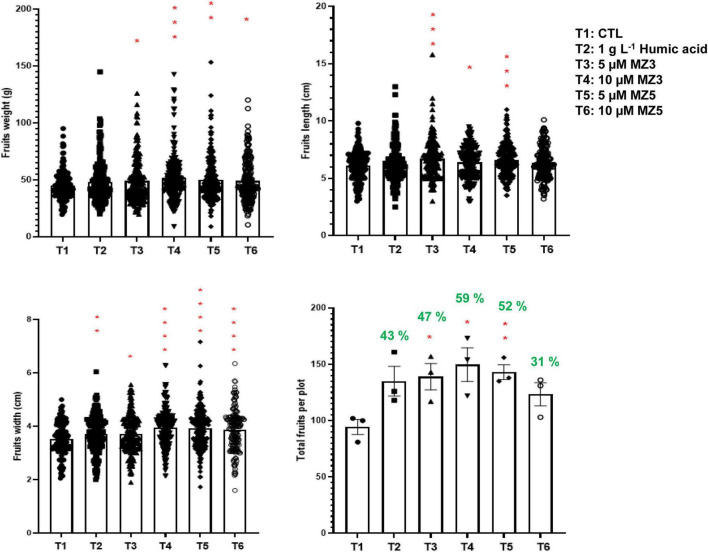
MiZax effect on green pepper fruit production from the KAUST field, performed in 2021. Each data point represents one plant (*n* ≥ 166). Data represent mean ± *SD*. Statistical analysis was performed using the two-tailed Student’s *t*-test. Asterisks indicate statistically significant differences as compared to CTL (*p < 0.05, **p < 0.01, ***p < 0.001, ****p < 0.0001). Symbols (•, CTL; ■, 1.5 g l^–1^ HA; ▲, 5 μM MZ3; ▼, 10 μM MZ3; ◆, 5 μM MZ5; and ○, 10 μM MZ5) used over error bars represent each replicates in the investigated group. CTL, Control; HA, humic acid; MZ3, MiZax3; MZ5, MiZax5.

To verify our findings in the KAU field, we further conducted another trial in 2021 at the King Abdullah University of Science and Technology (KAUST) experimental field, a warmer and more moisture place next to the Red Sea. Following the study design in the KAU, we compared 1 g l^–1^ of humic acid to 5 and 10 μM of MiZax. Constantly, the plant height, branches, flowers, and fruits of the green pepper were substantially increased after 60 days of planting (Figure 6), while the number of leaves showed high variability, which was due to windy climate. Furthermore, the weight, length, and width of harvested fruits were also considerably enhanced by MiZax (Figure 7), compared to humic acid and untreated control plot. Most importantly, MiZax caused 31–59% increase in total yield per plant (Figure 7). However, we observed in this trial that the lower concentration of MiZax5 had a better effect than 10 μM, which might suggest that higher concentrations of MiZax5 could not be optimal for plant growth and development. Although MiZax did not disturb the arbuscular mycorrhiza fungi spore germination (Wang et al., 2020), the safe application range of MiZax should be further studied.

### Mimics of Zaxinone Treatment Increased Squash Production in the Field

**FIGURE 8:**
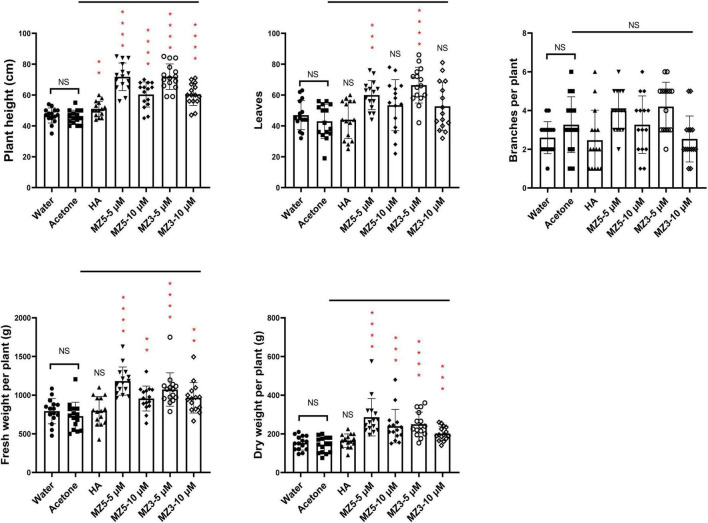
Plant phenotypical evaluation of MiZax on squash from the field of the KAU, performed in 2021. MiZax effect on crop production from the field of the KAU in 2021 (*n* = 15). Statistical analysis was performed using the two-tailed Student’s *t*-test. Asterisks indicate statistically significant differences as compared to CTL (**p < 0.01, ***p < 0.001, ****p < 0.0001). Symbols (•, CTL; ■, Acetone; ▲,1.5 g L^−1^ HA; ▼, 5 μM MZ5; ◆, 10 μM MZ5; ○, 5 μM MZ3; ◊ 10 μM MZ3) used over error bars represent each replicates in the investigated group. CTL, Control; HA, humic acid; MZ3, MiZax3; MZ5, MiZax5.

**FIGURE 9:**
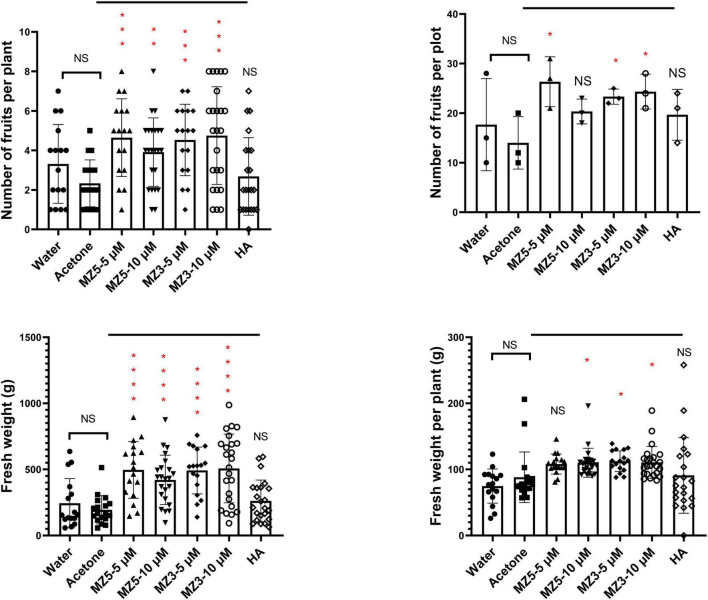
MiZax effect on squash fruit production from the field of the KAU, performed in 2021 (*n* ≥ 16). Statistical analysis was performed using the two-tailed Student’s *t*-test. Asterisks indicate statistically significant differences as compared to CTL (*p < 0.05, **p < 0.01, ***p < 0.001, ****p < 0.0001). Symbols (•, CTL; ■, Acetone; ▲, 5 μM MZ5; ▼, 10 μM MZ5; ◆, 5 μM MZ3; ○, 10 μM MZ3; and ◊, 1.5 g l^–1^ HA) used over error bars represent each replicates in the investigated group. CTL, Control; HA, humic acid; MZ3, MiZax3; MZ5, MiZax5.

Finally, to explore the suitability to other horticultural crops, we performed a trial on squash in the KAU field. Again, MiZax significantly boosted the height and biomass of squash plants, while humic acid failed to promote growth (Figure 8). Although no difference in the branches, the leaves number was observed following the low concentration of MiZax treatments (Figure 8). Surprisingly, only MiZax application significantly triggered the fruit production and weight, no matter per plant or plot, whereas humic acid did not show expected activity (Figure 9). We also observed that the low concentration of both MiZax had better bioactivity than the higher one, which indicates that MiZax should be applied in lower amounts in future studies. Additionally, the acetone mock did not show any difference compared to the water control group (Figures 8, 9), which suggests that the solvent used to dissolve MiZax did not play a significant role in these studies.

## Conclusion

In conclusion, based on our three independent field studies, the amount of applied MiZax was much lower (at the micromolar or mg level) than the commercial biostimulant humic acid with a better bioactivity in promoting crop plant growth and development. Thus, MiZax are potential biostimulants enhancing the performance and production of horticultural plants (monocots and dicots).

## Data Availability Statement

The original contributions presented in the study are included in the article/Supplementary Material, further inquiries can be directed to the corresponding author.

## Author Contributions

SA-B, IB, SA-S, and MM proposed the concept. JYW, MJ, and Md GH designed the experiments. JYW, MJ, Md GH, G-TEC, and LB conducted the experiments. JYW, MJ, Md GH, IB, MM, and SA-B analyzed and discussed the data. TO and TA synthesized and provided MiZax3 and MiZax5. JYW, MJ, and SA-B wrote the manuscript. All the authors have read, edited, and approved the final version of the manuscript.

## Conflict of Interest

The authors declare that the research was conducted in the absence of any commercial or financial relationships that could be construed as a potential conflict of interest.

## Publisher’s Note

All claims expressed in this article are solely those of the authors and do not necessarily represent those of their affiliated organizations, or those of the publisher, the editors and the reviewers. Any product that may be evaluated in this article, or claim that may be made by its manufacturer, is not guaranteed or endorsed by the publisher.

## Footnotes

1
https://www.isaaa.org/kc/cropbiotechupdate/article/default.asp?ID=4778
