# Supplementary material for: Evaluation of the Biostimulant Activity of Zaxinone Mimics (MiZax) in Crop Plants
Source: Front Plant Sci. 2022 Jun 16;13:874858. doi: 10.3389/fpls.2022.874858 (PMC9245435; doi:10.3389/fpls.2022.874858)
Supplement: Supplementary file 1 [file Data_Sheet_1.PDF]

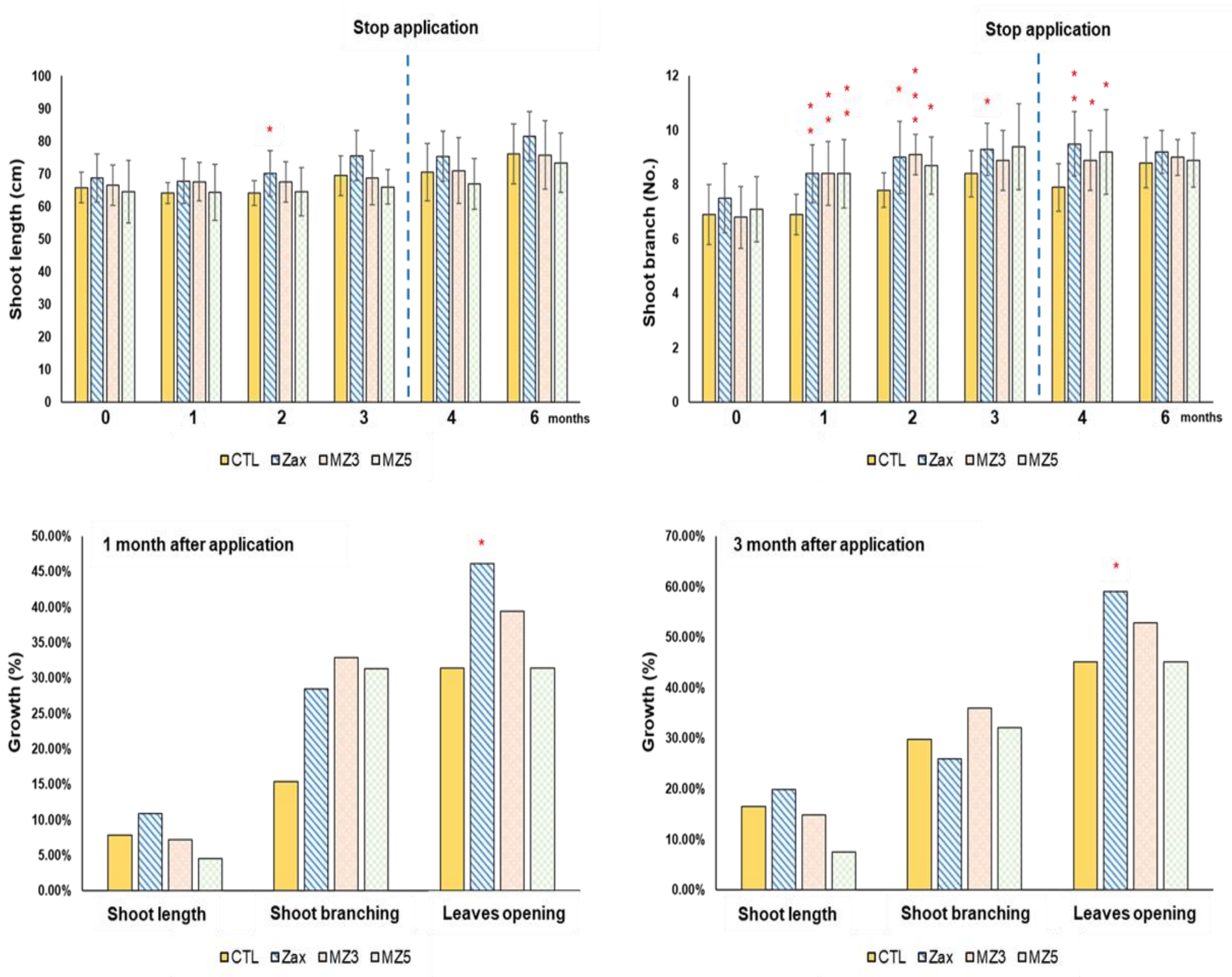

Supplementary Figure 1. Post-application effect of Zax, MZ3, and MZ5 at 5 µM on date palm plants under field conditions, performed in 2021. Data represent mean ± SD. n=10. Statistical analysis was performed using two-tail student t-test. Asterisks indicate statistically significant differences as compared to CTL (*$p < 0.05$, **$p < 0.01$, ***$p < 0.001$, ****$p < 0.0001$). CTL, Control; Zax, Zaxinone; MZ3, MiZax3; MZ5, MiZax5.

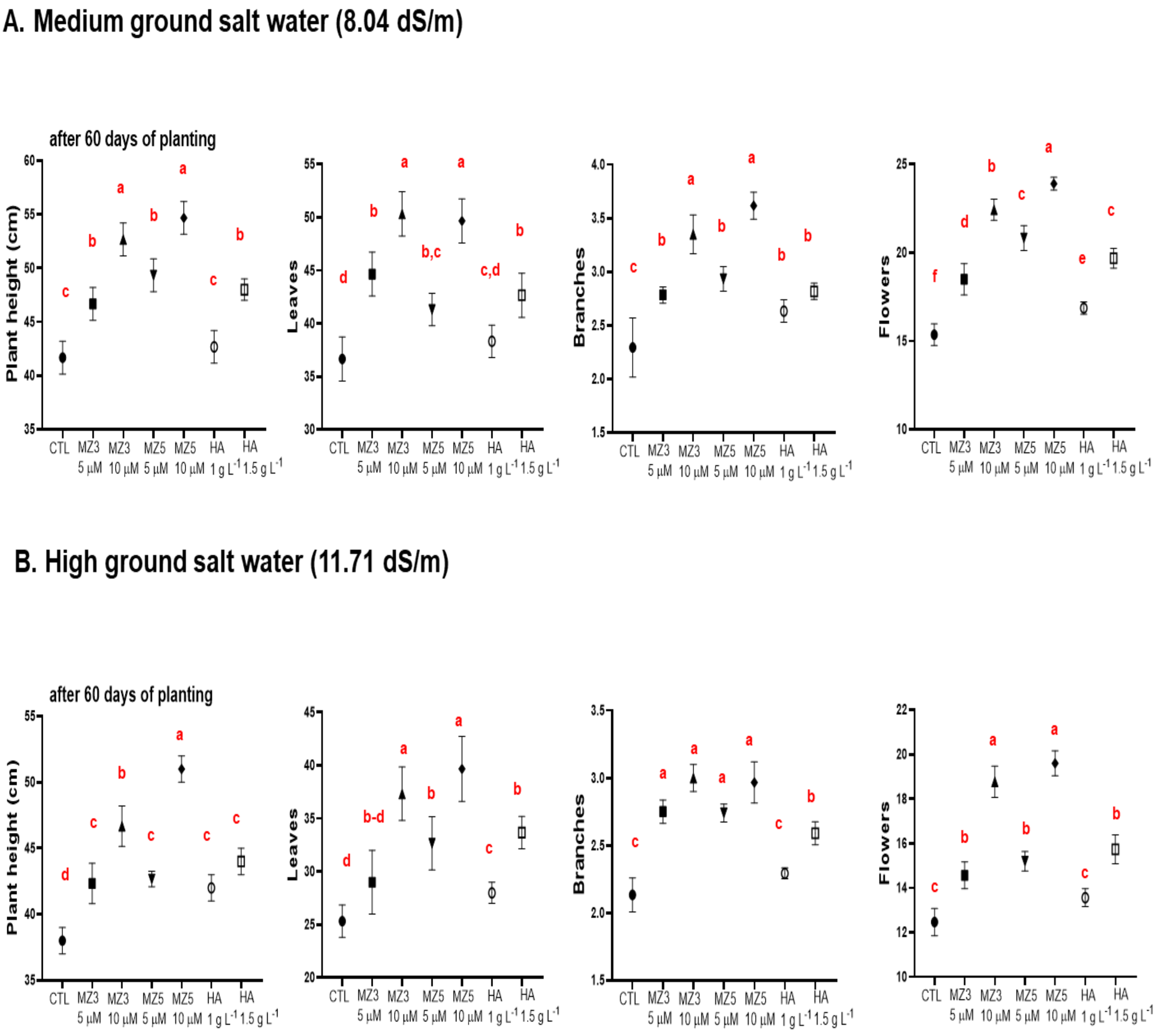

Supplementary Figure 2. Plant phenotypical evaluation of MiZax on green pepper grown under salty water conditions from the field of KAU in 2020. The data represents as the distribution of 15 plants from three plots (n=3). Data represent mean ± SD. Statistical analysis was performed using One-way analysis of variance (ANOVA) and Tukey's post hoc test. Different letters denote significant differences ($p < 0.05$). Symbols (●, CTL; ■, 5 μM MZ3; ▲, 10 μM MZ3; ▼, 5 μM MZ5; ♦, 10 μM MZ5; ○, 1 g L-1 HA; □, 1.5 g L-1 HA) used here represent each investigated group. HA, humic acid; MZ3, MiZax3; MZ5, MiZax5.

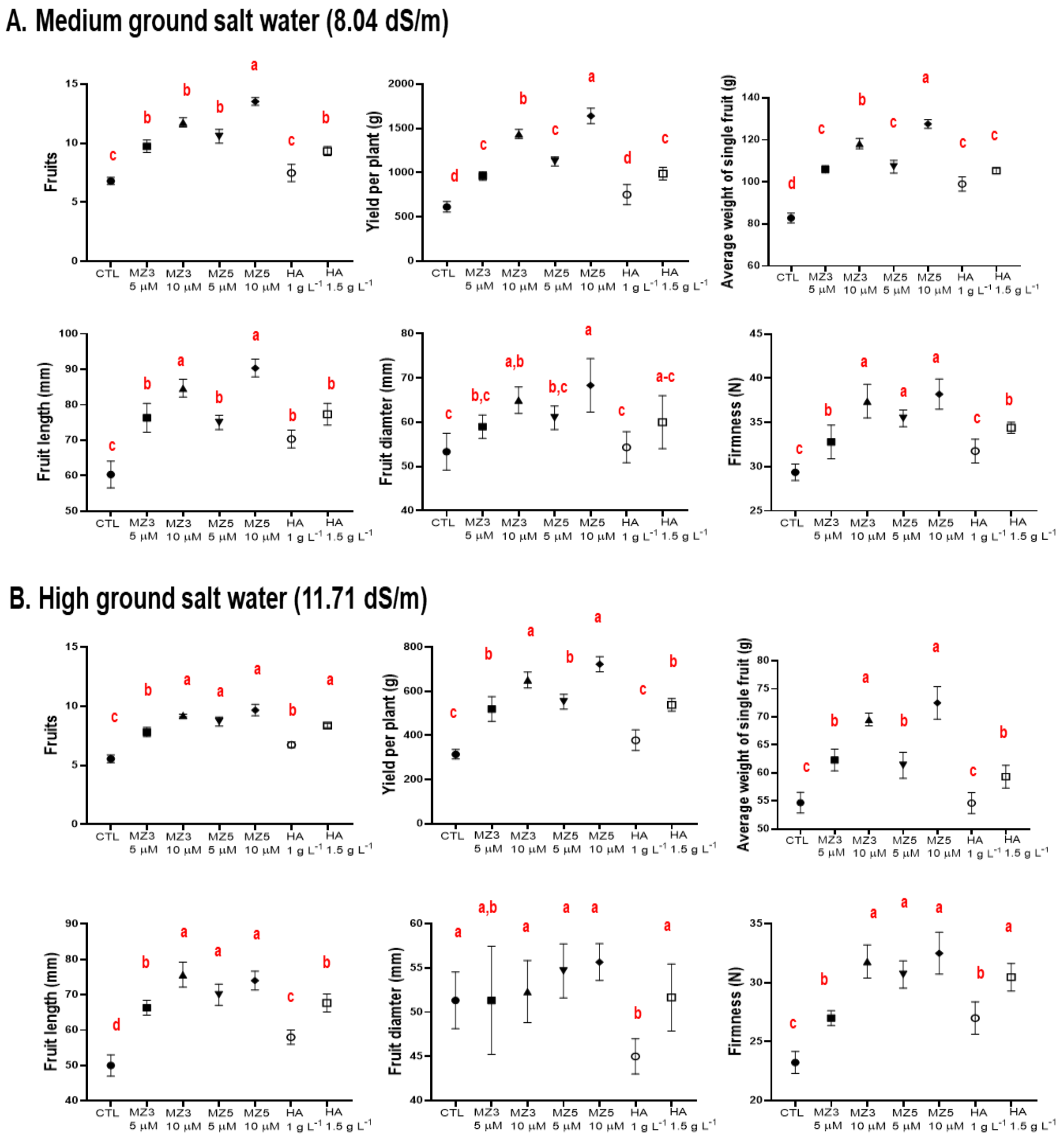

Supplementary Figure 3. MiZax effect on green pepper fruit production from the field of KAU in 2020 under salty water conditions. The data represents as the distribution of 15 plants from three plots (n=3). Data represent mean ± SD. Statistical analysis was performed using One-way analysis of variance (ANOVA) and Tukey's post hoc test. Different letters denote significant differences (p < 0.05). Symbols (●, CTL; ■, 5 μM MZ3; ▲, 10 μM MZ3; ▼, 5 μM MZ5; ♦, 10 μM MZ5; ○, 1 g L-1 HA; □, 1.5 g L-1 HA) used here represent each investigated group. HA, humic acid; MZ3, MiZax3; MZ5, MiZax5.

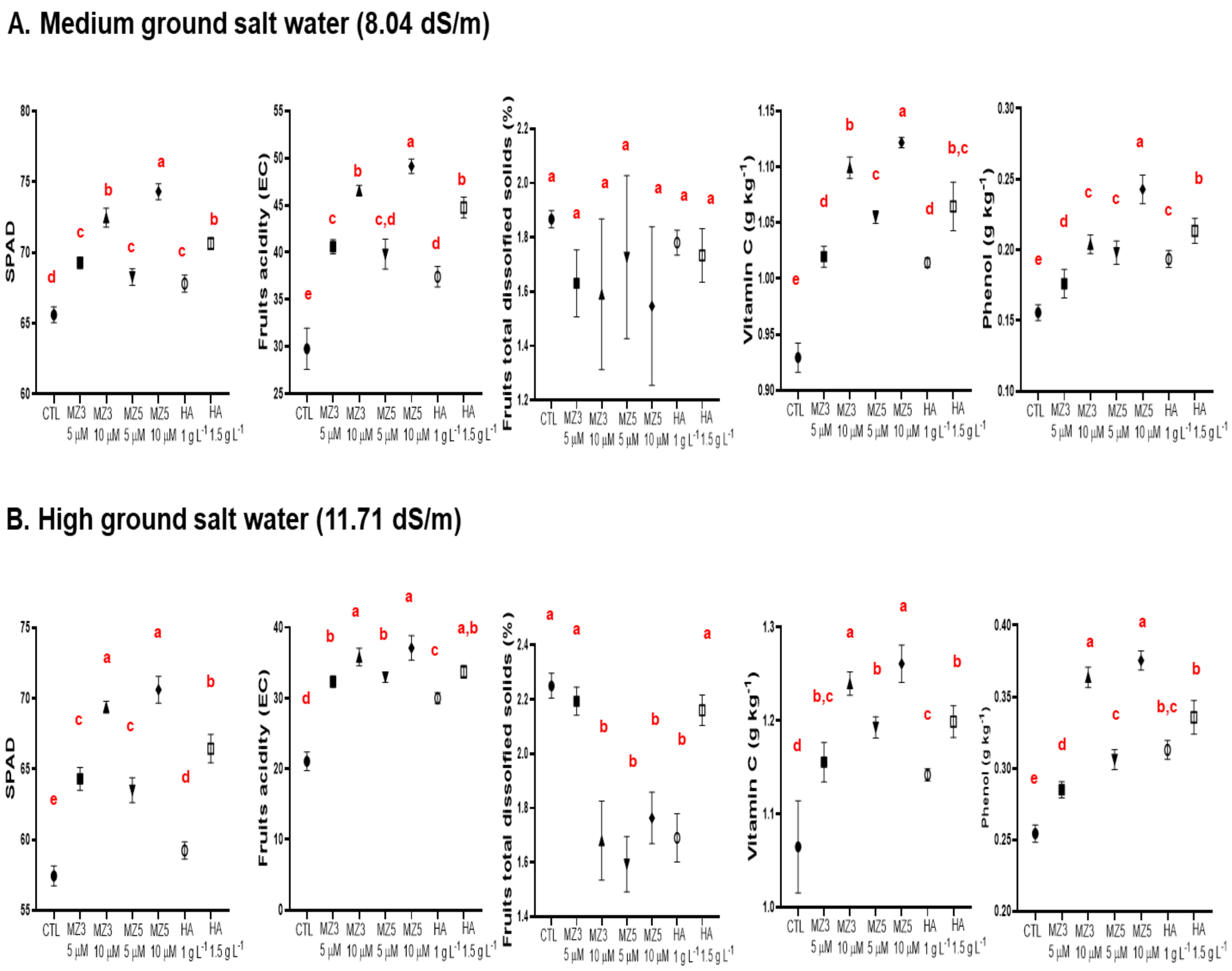

Supplementary Figure 4. Biochemical characterization of MiZax on green pepper grown under salty water conditions from the field of KAU in 2020. The data represents as the distribution of 15 plants from three plots (*n*=3). Data represent mean ± SD. Statistical analysis was performed using One-way analysis of variance (ANOVA) and Tukey's post hoc test. Different letters denote significant differences ($p < 0.05$). Symbols (●, CTL; ■, 5 μM MZ3; ▲, 10 μM MZ3; ▼, 5 μM MZ5; ♦, 10 μM MZ5; ○, 1 g L-1 HA; □, 1.5 g L-1 HA) used here represent each investigated group. HA, humic acid; MZ3, MiZax3; MZ5, MiZax5.
